# CD24 Is Not Required for Tumor Initiation and Growth in Murine Breast and Prostate Cancer Models

**DOI:** 10.1371/journal.pone.0151468

**Published:** 2016-03-15

**Authors:** Natascha Cremers, Antje Neeb, Tanja Uhle, Arno Dimmler, Melanie Rothley, Heike Allgayer, Riccardo Fodde, Jonathan Paul Sleeman, Wilko Thiele

**Affiliations:** 1 University of Heidelberg, Medical Faculty Mannheim, Mannheim, Germany; 2 Karlsruhe Institute of Technology, Institut für Toxikologie und Genetik, Karlsruhe, Germany; 3 Institut und Gemeinschaftspraxis für Pathologie an den St. Vincentiuskliniken Karlsruhe, Karlsruhe, Germany; 4 Department of Pathology, Erasmus MC Cancer Institute, Rotterdam, The Netherlands; University of Tennessee Health Science Center, UNITED STATES

## Abstract

CD24 is a small, heavily glycosylated, GPI-linked membrane protein, whose expression has been associated with the tumorigenesis and progression of several types of cancer. Here, we studied the expression of CD24 in tumors of MMTV-PyMT, *Apc*^1572/T+^ and TRAMP genetic mouse models that spontaneously develop mammary or prostate carcinoma, respectively. We found that CD24 is expressed during tumor development in all three models. In MMTV-PyMT and *Apc*^1572T/+^ breast tumors, CD24 was strongly but heterogeneously expressed during early tumorigenesis, but decreased in more advanced stages, and accordingly was increased in poorly differentiated lesions compared with well differentiated lesions. In prostate tumors developing in TRAMP mice, CD24 expression was strong within hyperplastic lesions in comparison with non-hyperplastic regions, and heterogeneous CD24 expression was maintained in advanced prostate carcinomas. To investigate whether CD24 plays a functional role in tumorigenesis in these models, we crossed CD24 deficient mice with MMTV-PyMT, *Apc*^1572T/+^ and TRAMP mice, and assessed the influence of CD24 deficiency on tumor onset and tumor burden. We found that mice negative or positive for *CD24* did not significantly differ in terms of tumor initiation and burden in the genetic tumor models tested, with the exception of *Apc*^1572T/+^ mice, in which lack of CD24 reduced the mammary tumor burden slightly but significantly. Together, our data suggest that while CD24 is distinctively expressed during the early development of murine mammary and prostate tumors, it is not essential for the formation of tumors developing in MMTV-PyMT, *Apc*^1572T/+^ and TRAMP mice.

## Introduction

CD24, also known as heat stable antigen (HSA) or CD24a in the mouse, is a small GPI-linked membrane glycoprotein. Consisting of only around 30 amino acids [1–3], CD24 is heavily N- and O-glycosylated, and has a molecular weight of 30 to 70 kDa, depending on the state of glycosylation [4].

CD24 is expressed by cells of the hematopoietic and immune systems [5–7], in the developing brain, kidney, prostate and mammary gland [8–11], and in gastrointestinal cells such as Paneth cells and parietal cells [12,13]. Functionally it has been implicated in hematopoiesis and immune cell function [14,15], neurite outgrowth [16,17], clonal expansion of T-cells [18] and mammary gland development [11]. Consistently, CD24 deficient mice exhibit quantitative and qualitative alterations in several types of immune cell [19–21], increased neurogenesis [22], transiently enhanced branching morphogenesis in mammary glands [11], and modified gastrointestinal pathophysiology [13,23].

A broad literature associates CD24 expression with the development and progression of human tumors. Upregulated CD24 expression has been reported for many types of human cancer [24], and its expression correlates significantly with different clinicopathologic properties [25–30]. Furthermore, CD24 expression has been shown to have prognostic relevance for the survival of patients suffering from a variety of cancer types, including breast and prostate [28–32]. Patient survival is closely linked to metastasis [33]. Accordingly, we and others have shown that CD24 can promote metastasis in a number of ways [24,34–39].

CD24 expression has been shown to be upregulated during tumorigenesis in gastric, colon and breast cancer patients [27,40], suggesting that besides its role in tumor progression and metastasis, CD24 may also play a role in early tumor development. To determine if this is the case, we examined here the expression of CD24 during autochthonous tumorigenesis in the mouse mammary tumor virus-polyoma middle T (MMTV-PyMT) and adenomatous polyposis coli (*Apc*^1572T/+^) genetic mouse models of breast cancer, as well as in the transgenic adenocarcinoma of mouse prostate (TRAMP) cancer model, and determined the effect of CD24 deficiency on tumorigenesis in these models. MMTV-PyMT mice express the mammary tumor virus-polyoma middle T (PyMT) oncoprotein under the control of mouse mammary tumor virus LTR (MMTV LTR) [41]. Thus, PyMT expression is restricted to mammary epithelium, and induces the malignant transition from benign lesions to invasive carcinoma in distinct stages (hyperplasia, adenoma/mammary intraepithelial neoplasia, early carcinoma, late carcinoma) with subsequent metastasis, thereby recapitulating the development of luminal-like human breast cancer [42,43]. *Apc*^1572T/+^ mice carry a mutated *Apc* gene encoding for a protein truncated at position 1572, resulting in a predisposition to the development of multifocal basal-like mammary tumors that resemble human metaplastic carcinoma [44]. TRAMP mice develop prostate tumors that phenocopy the pathogenesis of the human disease due to transgenic expression of the SV40 large T antigen under control of the probasin promoter [45,46]. The studies we present here suggest that although CD24 is expressed during murine mammary and prostate tumorigenesis, its genetic ablation does not affect tumor formation and growth in either model.

## Materials and Methods

### Immunohistochemistry

Mammary glands, prostates and seminal vesicles were embedded in cryosectioning medium, and 8–10 μm sections were prepared. The sections were then fixed in cold acetone, blocked with 10% goat serum/1% rabbit serum in PBS, and a biotin blocking kit (DAKO, Hamburg, Germany), and incubated with primary antibodies directed against CD24 (Becton Dickinson, Heidelberg, Germany) over night at 4°C. Binding of the primary antibody was visualised using biotin-coupled secondary antibodies and an alkaline-phosphatase-complex (DAKO). Fuchsin was used as a chromogenic substrate. The sections were counterstained with hematoxylin, and analysed using an Axioskop microscope (Zeiss, Jena, Germany) equipped with an Axiocam camera (Zeiss), and Axiovision software (Zeiss). Histopathological analysis was performed, and the intensity of the CD24 staining was evaluated using the following scoring system:—no staining; + moderate staining; ++ strong staining. To test the null hypothesis "staining intensity is independent of histopathologic appearance", two-sided Fisher´s exact tests were performed.

### Experimental mice and genotyping

Mice were maintained on a C57BL/6 background and genotyped as follows. For *Apc*^1572T/+^ mice, primers APC-C2, APC-A3 and APC-pN3 were applied in a single PCR reaction that amplified a 180 bp fragment for the wild type *Apc* gene, and an additional 250 bp fragment in the case of heterozygous mutant mice. Transgenic MMTV-PyMT offspring were identified using PymT3p and PymT4m primers to amplify a 160 bp fragment of the PyMT transgene, and Plg-in2-3' and Plg-ex2-5' primers to amplify a 268 bp fragment of the plasminogen precursor gene as an internal control. B6,Tg(TRAMP)8247Ng(T/+) mice were genotyped with Tag rev and PB1 for primers to amplify the transgene (600 bp fragment), while amplification of a 500 bp casein fragment with mCasein forward and mCasein reverse primers served as an internal control. The presence of knockout or wild-type alleles of the *CD24* gene was assessed by using three primers (wt fw, KO rev and 5’neo rev) in a single reaction. The amplified products were 300 bp for the wild-type allele and 550 bp for the knockout allele.

Primer sequences were as follows:

APC-C2 (5’-GGAAAAGTTTATAGGTGTCCCTTCT-3’)

APC-A3: (5’-CTAGCCCAGACTGCTTCAAAAT-3’)

APC-pN3: (5’-GCCAGCTCATTCCTCCACTC-3’)

PymT3p: (5’-CGGCGGAGCGAGGAACTGAGGAGAG-3’)

PymT4m: (5’-TCAGAAGACTCGGCAGTCTTAGGCG-3’)

Plg-in2-3': (5’-TGTGGGCTCTAAAGATGGAACTCC-3’)

Plg-ex2-5': (5’-GACAAGGGGACTCGCTGGATGGCTA-3’)

mCasein forward: 5’-GATGTGCTCCAGGCTAAAGTT-3’

mCasein reverse: 5’-AGAAACGGAATGTTGTGGAGT-3’

Tag rev: 5’-CTCCTTTCAAGACCTAGAAGGTCCA-3’

PB1for: 5’-CCGGTCGACCGGAAGCTTCCACAAGTGCATTTA-3’

mCasein forward: 5’-GATGTGCTCCAGGCTAAAGTT-3’

mCasein reverse: 5’-AGAAACGGAATGTTGTGGAGT-3’

wt fw: 5’-AGCGGACATGGGCAGAGCGATGGTGG-3’

KO rev: 5’-GTGGTTCGCAGGGAGCGCGAAGACCTC-3’

5’neo rev: 5’-TGACAGCCGGAACACGGCGGCATCAGA-3’

### Animal experiments

In experiments where MMTV-PyMT, *Apc*^1572T/+^, and TRAMP mice were crossed with *CD24*^-/-^ mice [19], animals heterozygous for the tumor-initiating transgene/mutation and either *CD24*^-/-^ or *CD24*^+/+^ were used. To assess the effect of CD24 deficiency on tumor development in MMTV-PyMT, *Apc*^1572T/+^ models, female mice that were either *CD24*^-/-^ or *CD24*^+/+^ were palpated weekly to establish the age at which mammary tumors could first be detected. Following tumor detection, tumors were measured twice a week. When one tumor reached 1 cm in diameter in one dimension, animals were sacrificed, tumors and mammary tissue were removed in total and weighed. Male TRAMP mice that were either *CD24*^-/-^ or *CD24*^+/+^ were sacrificed at the fixed time point of 6 months of age. Prostate and seminal vesicles were then excised and weighed.

All animal experiments were performed according to German legal requirements. In accordance with the German law that was active at the time when the experiments were performed, an extra approval by the local authorities was not necessary, as we used tumor-prone genetic animal models that were not subjected to any treatment or other intervention. Permission to breed, house and sacrifice animals (§11) was granted to the animal facility of the Institute of Toxicology and Genetics, Karlsruhe Institute of Technology, by the Regierungspräsidium Karlsruhe (AZ35-9185.64). The animals were sacrificed by cervical dislocation, as requested by the German animal work guidelines for mice. All researchers (NC (zoologist; registration date: 02.09.2009) AN (AZ35-9185.83_09.07.2008) JPS (AZ35-9185.83_20.05.1998) WT (zoologist; registration date: 15.05.2008)) and animal facility members (Selma Huber (AZ35-9185.83_12.01.2007), Manuela Sauer (AZ35-9185.83_28.01.2010)) that were involved in animal work for this study have been registered or authorized by the Regierungspräsidium Karlsruhe (§9). According to the German legal requirements zoologists (NC, WT) need no extra permission to kill animals, they need only to be registered at the Regierungspräsidium, which was done at the indicated dates.

## Results

### CD24 is expressed in MMTV-PyMT and *Apc*^1572T/+^ mammary tumors, and CD24 expression correlates with differentiation grade of the lesions

The normal expression pattern of CD24 in the non-transformed mammary gland has been documented [11], and the stages of mammary tumorigenesis in the MMTV-PyMT model [42] and *Apc*^1572T/+^ model [44] have been previously described. To assess the expression pattern of CD24 in these two breast cancer models, female animals of different ages were sacrificed, and their mammary glands analysed by staining sections immunohistochemically for CD24.

**Fig 1:**
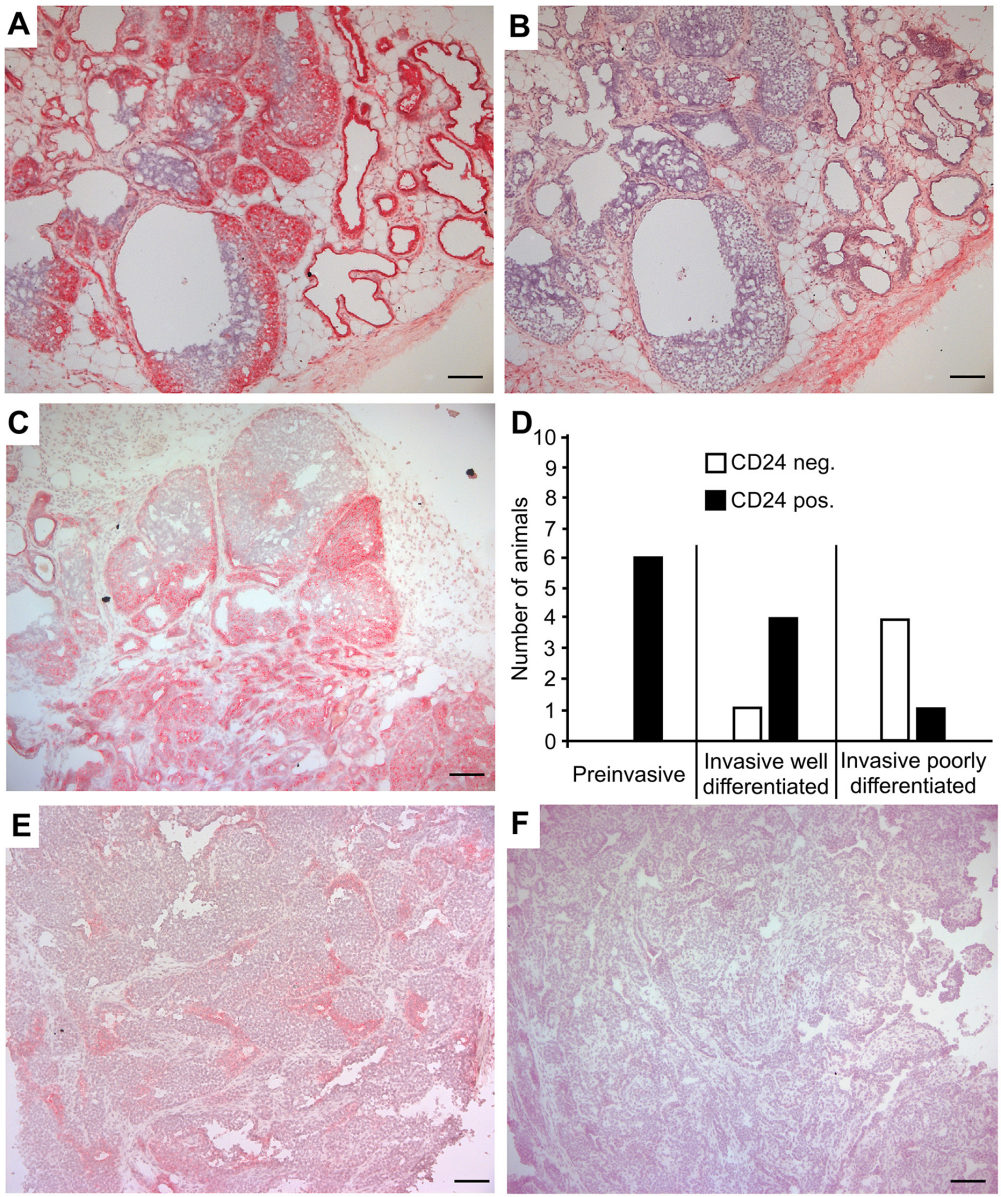
CD24 is expressed during MMTV-PyMT mammary tumorigenesis. To determine the expression of CD24 during tumorigenesis, female MMTV-PyMT mice were sacrificed at a variety of ages, and their mammary glands were cut into sections and stained with antibodies specific for CD24. After counterstaining with hematoxylin, the sections of 12 animals were photographed and analysed. Representative sections are shown. Scale bars indicate 100 μm. (A) Hyperplastic preneoplastic lesions and small adenomas that are either stained strongly or negative for CD24. (B) Isotype control stained serial section corresponding to A. (C) Different degrees of CD24 staining within one and the same neoplastic lesion. (D) A histopathologic analysis was performed and the intensity of the CD24 staining was evaluated. Score:—no staining; + moderate staining; ++ strong staining. A two-sided Fisher´s exact test was performed to test the null hypothesis "staining intensity is independent of histopathologic appearance". The null hypothesis was rejected based on a calculated p-value of 0.00035 (3x3 contingency table). Scoring was categorized into CD24 negative ("-") or CD24 positive ("+" or "++"), and two-sided Fisher´s exact tests and 2x2 contingency tables were used to perform pairwise comparisons of (i) "invasive well differentiated" vs. "invasive poorly differentiated" (p = 0.21), (ii) "preinvasive" vs. "invasive well differentiated" (p = 0.45) and (iii) "preinvasive" vs. "invasive poorly differentiated" (p = 0.015). (E) More advanced tumor showing weak and diffuse staining. **F:** More advanced tumor negative for CD24.

In the MMTV-PyMT model we observed that hyperplastic pre-neoplastic lesions and small adenomas stained strongly for CD24 (Fig 1A), reflecting expression in the normal mammary epithelium, where it is predominantly present in the luminal epithelium and only weakly expressed in basal myoepithelial cells [11]. However, heterogeneity was observed in the distribution of the stained cells, with some hyperplastic lesions being stained strongly, whereas others were negative (Fig 1A). Furthermore, different degrees of staining or areas of CD24 positivity were often present within one and the same neoplastic lesion, with some parts being stained whereas other parts were not (Fig 1C). The expression levels of CD24 were increased in poorly differentiated *versus* well-differentiated lesions and correlated with tumor grade in a statistically significant manner (Fig 1D; S1 Table). Accordingly, as tumors progressed and increased in size, staining intensity generally decreased, and more advanced tumors showed either weak and diffuse staining (Fig 1E) or were completely negative for CD24 (Fig 1F).

**Fig 2:**
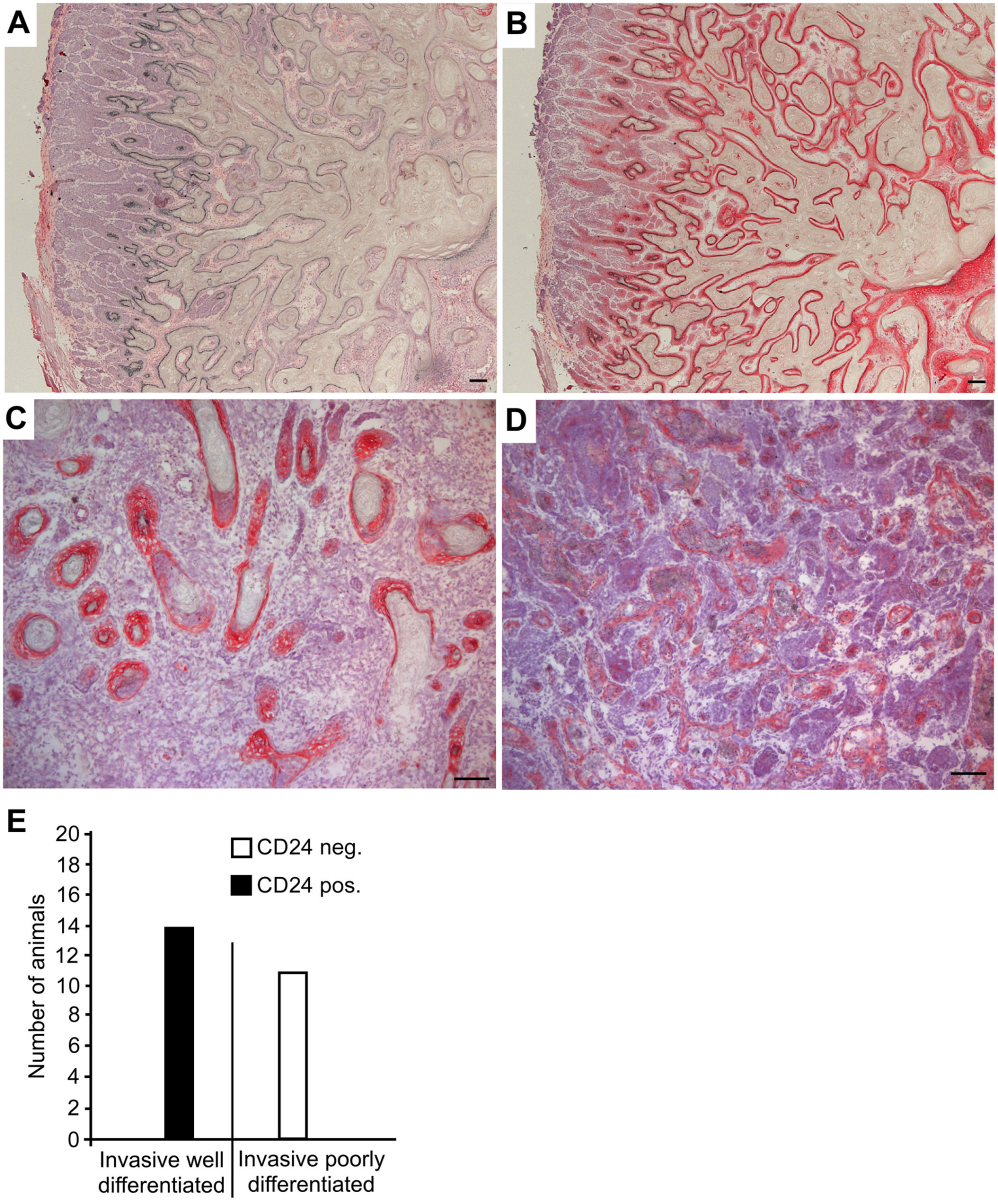
CD24 is expressed in mammary tumors during *Apc*^*1572/T+*^ tumorigenesis. To determine the expression of CD24 during tumorigenesis, female *Apc*^*1572/T+*^ mice were sacrificed at a variety of ages, and their mammary glands were cut into sections and stained with antibodies specific for CD24. After counterstaining with hematoxylin, the sections of 14 animals were photographed and analysed. Representative sections are shown. Scale bars indicate 100 μm. (A) Isotype control stained serial section corresponding to (B). (B), (C) Epithelial layers surrounding the fibrillar deposits found in squamous metaplasia staining strongly for CD24. (D) Adenocarcinoma staining weakly for CD24. (E) A histopathologic analysis was performed and the intensity of the CD24 staining was evaluated. Score:—no staining; + moderate staining; ++ strong staining. A two-sided Fisher´s exact test was performed to test the null hypothesis "staining intensity is independent of histopathologic appearance". The null hypothesis was rejected based on a calculated p-value of 0.0000002 (2x2 contingency table).

Immunohistochemical analysis of *Apc*^1572T/+^ tumors reflected the squamous metaplasia morphology described previously [44]. CD24 staining was prominent and intense in the epithelial layers surrounding the fibrillar deposits found in the squamous metaplasia (Fig 2A and 2C), while staining was weaker in adenocarcinomas (Fig 2D). We found a strong and statistically significant correlation between differentiation grade (poorly and well differentiated, respectively) and staining intensity (Fig 2E; S2 Table).

### Lack of CD24 does not affect tumorigenesis in MMTV-PyMT and *Apc*^1572T/+^ breast cancer models significantly, but reduces mammary tumor burden in *Apc*^1572T/+^ mice

To assess the effect of CD24 deficiency on mammary tumor development and growth, MMTV-PyMT and *Apc*^1572T/+^ mice that were either *CD24*^+/+^ or *CD24*^-/-^ were palpated weekly to establish the time at which mammary tumors could first be detected after tumor initiation. Although tumors are already initiated once they become palpable, this strategy is a well-established method to define tumor onset in living mice, and serves as a surrogate measure of tumor initiation. All animals developed tumors, regardless of presence or absence of CD24. Following tumor detection, tumors were measured twice a week. In many cases, multiple tumors developed in the same animal. Animals were sacrificed when one tumor reached 1 cm in diameter in one dimension. Tumors and mammary tissue were then removed in total and weighed.

**Fig 3:**
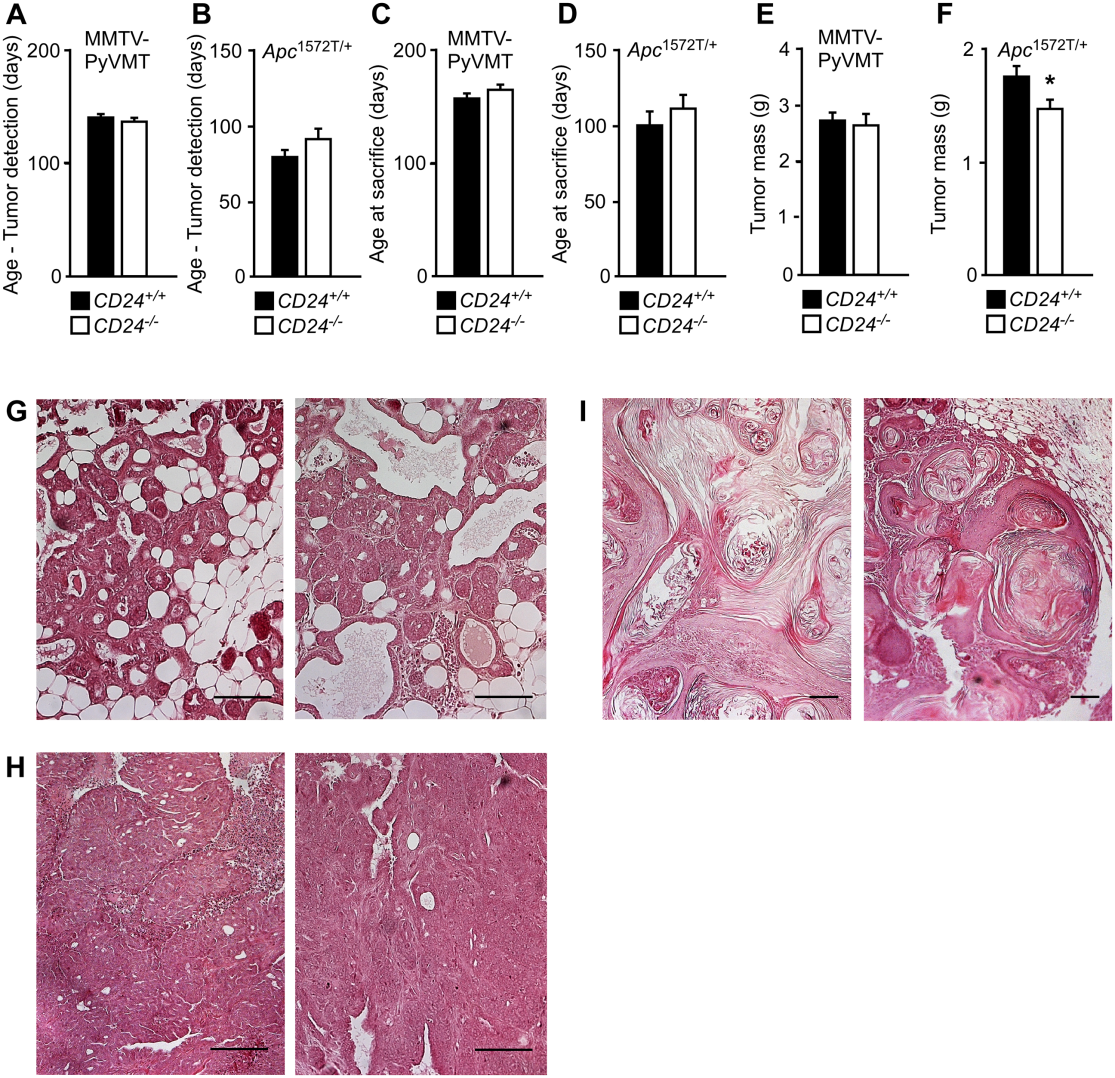
CD24 deficiency does not affect MMTV-PyMT mammary tumorigenesis, but reduces tumor burden in *Apc*^*1572/T+*^ mice. To study the effect of CD24 deficiency on mammary tumor initiation and growth, MMTV-PyMT and *Apc*^*1572/T+*^ mice were crossed with *CD24*^*-/-*^ mice. Female MMTV-PyMT and *Apc*^*1572/T+*^ mice either *CD24*^*-/-*^ or *CD24*^*+/+*^ were regularly palpated to monitor the time point of tumor onset. Following onset, tumors were measured regularly and animals were sacrificed when the tumors reached 1 cm in diameter in one dimension. Tumors and mammary tissue were then removed in total, and their mass was determined as a measure for tumor burden. Error bars indicate SE. Significance was tested using 2-tailed un-paired t tests assuming equal variance. Scale bars indicate 100 μm. (A) Age at which tumors were first detected in MMTV-PyMT mice; *CD24*^*+/+*^: n = 20; *CD24*^*-/-*^: n = 21. (B) Age at which tumors were first detected in *Apc*^*1572/T+*^ mice; *CD24*^*+/+*^: n = 19; *CD24*^*-/-*^: n = 20. (C) Age at which MMTV-PyMT mice were sacrificed; *CD24*^*+/+*^: n = 20; *CD24*^*-/-*^: n = 21. (D) Age at which *Apc*^*1572/T+*^ mice were sacrificed; *CD24*^*+/+*^: n = 19; *CD24*^*-/-*^: n = 21. (E) MMTV-PyMT tumor burden; *CD24*^*+/+*^: n = 20; *CD24*^*-/-*^: n = 21. **F:** *Apc*^*1572/T+*^ tumor burden; *CD24*^*+/+*^: n = 19; *CD24*^*-/-*^: n = 20 * p<0.05. (G), (H) Representative hematoxylin stained sections of MMTV-PyMT *CD24*^*+/+*^ (left panels) and MMTV-PyMT *CD24*^*-/-*^ (right panels) mammary tumors. Evaluation of the sections showed poorly differentiated mammary carcinoma in situ/carcinoma (G3) with additional accompanying earlier stage lesions (hyperplasia, papilloma, adenoma) present in virtually all tumors. There was no difference between *CD24*^*+/+*^ and *CD24*^*-/-*^ MMTV-PyMT mammary tumors with the exceptions that necrotic areas were slightly less frequent in *CD24*^*-/-*^ tumors. (I) Representative hematoxylin stained sections of *Apc*^*1572/T+*^ *CD24*^*+/+*^ (left panel) and *Apc*^*1572/T+*^ *CD24*^*-/-*^ (right panel) mammary tumors. Histopathologic evaluation of the sections showed metaplastic carcinoma with squamous cell-like differentiation (well-differentiated; G1). There was no obvious difference between *CD24*^*+/+*^ and *CD24*^*-/-*^ *Apc*^*1572/T+*^ mammary tumors.

CD24 deficiency had no significant effect on either the age at which tumors were first detected (Fig 3A and 3B), nor on the age at which the animals were sacrificed (Fig 3C and 3D). The effect of CD24 deficiency on the tumor burden was assessed by quantifying the mass of the tumor-bearing mammary glands. Tumor mass was statistically significantly different (p<0.05) between the *CD24*^+/+^ and *CD24*^-/-^ groups in *Apc*^1572T/+^ mice, although the difference was marginal (Fig 3F), whereas no effect could be observed in MMTV-PyMT animals. To exclude possible morphological differences between tumors derived from *CD24*^+/+^ or *CD24*^-/-^ animals, we analysed hematoxylin-stained sections of MMTV-PyMT and *Apc*^1572T/+^ tumors, but found no difference in the histological appearance of the tumors (Fig 3G–3I). Together, these data show that while CD24 is expressed in both models during tumor development, CD24 deficiency had no significant effect on the initiation of tumors and subsequently reduced tumor burden in a statistically significant manner only in *Apc*^1572T/+^ mice, albeit marginally.

### CD24 is expressed in the normal murine prostate, and during TRAMP prostate tumorigenesis

**Fig 4:**
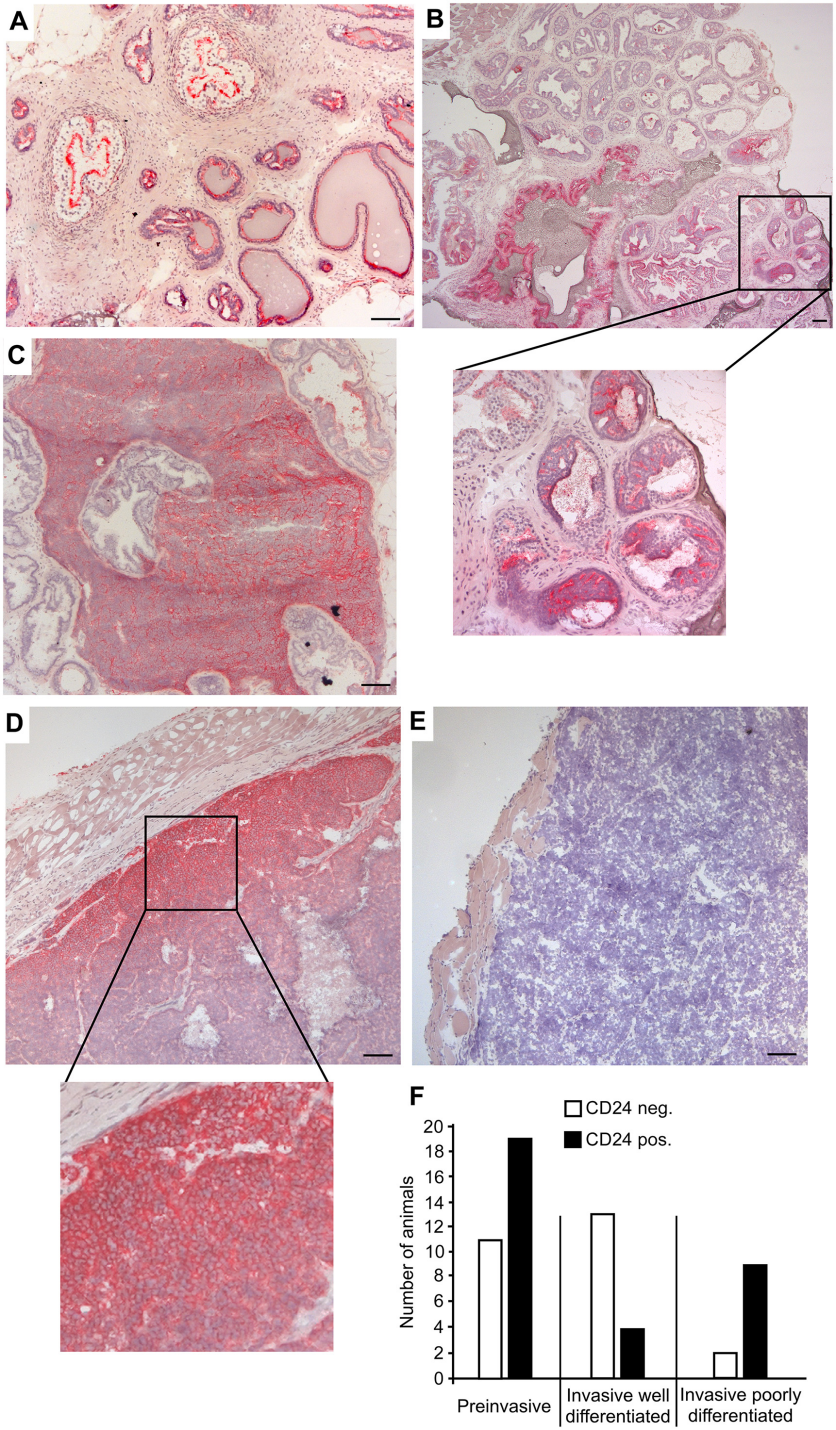
CD24 is expressed in prostate tumors during TRAMP tumorigenesis. Male TRAMP mice were sacrificed at a variety of ages, and their prostate glands were cut into sections and stained with antibodies specific for CD24. After counterstaining with hematoxylin, the sections of 52 animals were photographed and analysed. Representative sections are shown. Scale bars indicate 100 μm. (A) Prostate gland of a 12 week old C57BL/6 mouse showing the linings of the luminal layer of dorsolateral tubules staining positively for CD24. (B) Hyperplastic lesions in the prostate of a 12 week old TRAMP mouse with widespread but inhomogenous CD24 staining being stronger in hyperplastic areas, compared with adjacent non-hyperplastic regions. (C) Small TRAMP tumor staining homogenously and strongly for CD24. Larger TRAMP tumors were either CD24-positive (D) or negative (E). (F) A histopathologic analysis was performed and the intensity of the CD24 staining was evaluated. Score:—no staining; + moderate staining; ++ strong staining. A two-sided Fisher´s exact test was performed to test the null hypothesis "staining intensity is independent of histopathologic appearance". The null hypothesis was rejected based on a calculated p-value of 0,0000007 (3x4 contingency table). Scoring was categorized into CD24 negative ("-") or CD24 positive ("+" or "++"), and two-sided Fisher´s exact tests and 2x2 contingency tables were used to perform pairwise comparisons of (i) "invasive well differentiated" vs. "invasive poorly differentiated" (p = 0.006), (ii) "preinvasive" vs. "invasive well differentiated" (p = 0.015) and (iii) "preinvasive" vs. "invasive poorly differentiated" (p = 0.45).

To analyse CD24 expression during TRAMP prostate tumorigenesis, we immunostained prostate sections from mice of different ages for CD24. Hyperplastic lesions or PIN (prostate intraepithelial neoplasia) were observed starting from 12 weeks of age. In the normal prostate of age-matched non-transgenic C57BL/6 mice, prominent CD24 staining was found on the lining of the luminal layer of dorsolateral prostatic tubules (Fig 4A). However, in hyperplastic areas in TRAMP prostates, CD24 staining was stronger compared with adjacent non-hyperplastic areas (Fig 4B). CD24 staining in hyperplastic TRAMP lesions was generally widespread but heterogeneous with regard to distribution and intensity of the staining within a given tissue section (Fig 4B). Indeed, CD24-negative PINs were observed in areas of positively-stained lesions even within the same prostatic duct. CD24 staining was homogenous and strong in small prostate carcinomas (Fig 4C), although differences in intensity and distribution were apparent. Larger tumors were either CD24 positive (Fig 4D) or negative (Fig 4E). In the CD24-positive tumors, strong staining was found mainly at the periphery, with staining towards the necrotic centre being only weak or even absent. Although CD24 staining was heterogeneous, significantly fewer invasive well-differentiated tumors expressed CD24 compared to other tumor stages (Fig 4F; S3 Table).

### CD24 deficiency does not significantly influence tumorigenesis in TRAMP mice

Due to the internal development of prostate tumors, it was not possible to determine accurately when tumors first began to develop in the TRAMP model. To assess whether CD24 deficiency affects tumor development in this model, animals were therefore sacrificed at the fixed time point of 6 months of age. In addition to prostate tumors, TRAMP mice on the C57BL/6 background develop epithelial-stromal seminal vesicle tumors that reach an incidence of 100% at 6 months [47]. Both prostate and seminal vesicles were therefore excised and weighed separately when the animals were sacrificed.

**Fig 5:**
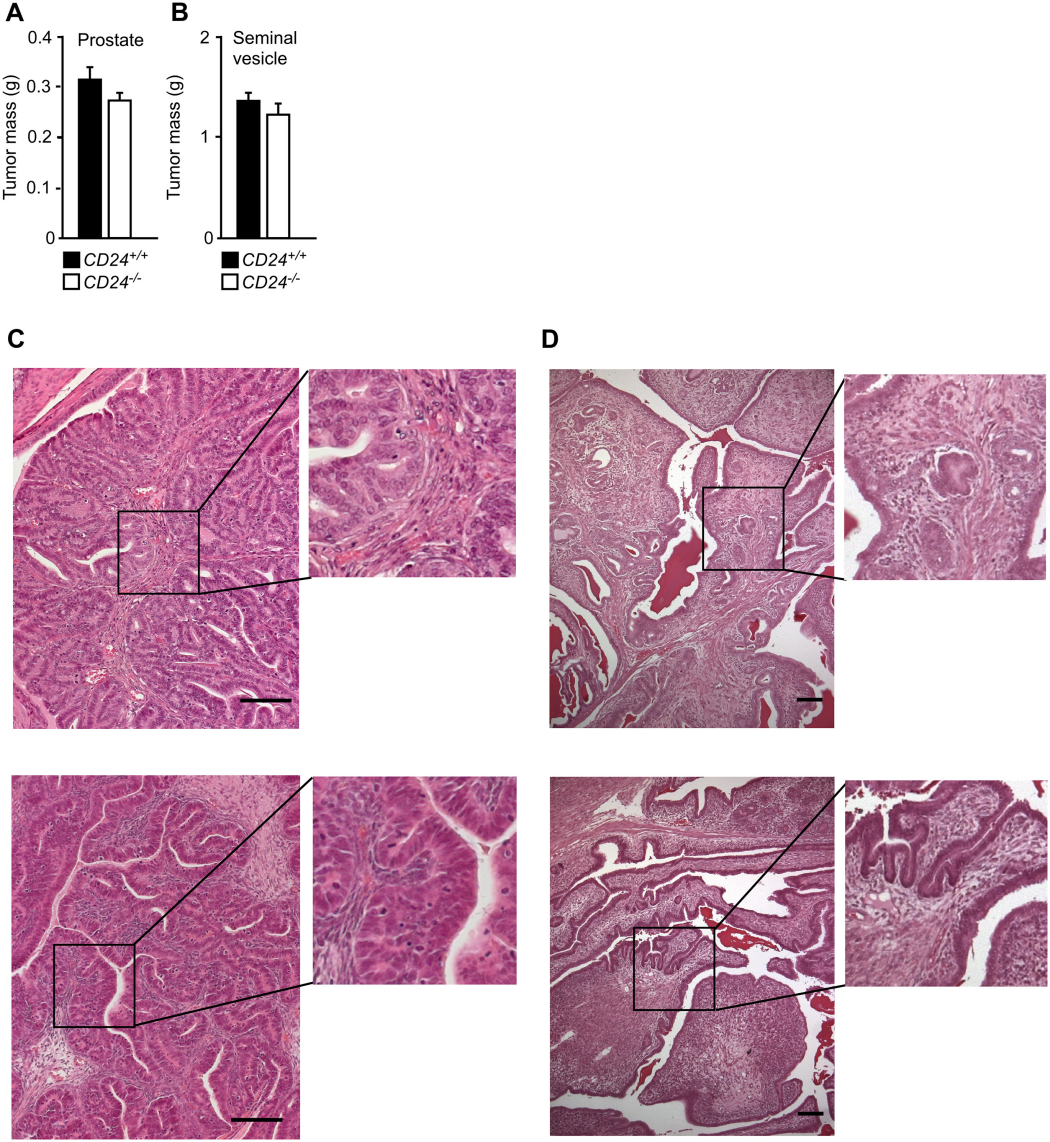
Lack of CD24 does not significantly affect TRAMP prostate and seminal vesicle tumor burden. To study the effect of CD24 deficiency on prostate tumorigenesis, TRAMP mice were crossed with *CD24*^*-/-*^ mice. Male TRAMP mice either ^-/-^ or ^+/+^ for *CD24* were sacrificed at 6 months of age. Prostate and seminal vesicles were then removed in total, and their mass was determined, as a measure for tumor burden. Error bars indicate SE. Significance was tested using 2-tailed un-paired t tests assuming equal variance. Scale bars indicate 100 μm. (A) Prostate tumor burden; *CD24*^*+/+*^: n = 23; *CD24*^*-/-*^: n = 21. (B) Seminal vesicle tumor burden; *CD24*^*+/+*^: n = 23; *CD24*^*-/-*^: n = 21. (C) Representative hematoxylin stained sections of *CD24*^*+/+*^ (upper panel) *CD24*^*-/-*^ (lower panel) neoplastic prostates. Histopathologic evaluation of the sections predominantly showed prostatic intraepithelial neoplasia (PIN) with small lesions developing into early invasive carcinoma. There was no difference between *CD24*^*+/+*^ and *CD24*^*-/-*^ prostate tumors. (D) Representative hematoxylin stained sections of *CD24*^*+/+*^ (upper panel) *CD24*^*-/-*^ (lower panel) neoplastic seminal vesicles. Evaluation of the sections showed dilated seminal vesicles with small tumor areals present. There was no difference between *CD24*^*+/+*^ and *CD24*^*-/-*^ seminal vesicle tumors.

Although there was a trend towards slightly reduced tumor burden in CD24-deficient animals, no significant difference in the burden of prostate or seminal vesicle tumors was observed (Fig 5A and 5B). There was also no difference in the morphology of tumors taken from *CD24*^-/-^ or *CD24*^+/+^ mice (Fig 5C and 5D). These data show that despite frequent and prominent expression of CD24 in TRAMP tumors, absence of CD24 did not significantly influence tumor burden.

## Discussion

Here, we show that in MMTV-PyMT, *Apc*^1572T/+^, and TRAMP mice, CD24 is prominently but heterogeneously expressed during early breast and prostate tumorigenesis. While CD24 expression inversely correlated with tumor grade, and thus generally decreased in progressive mammary tumors, it remained robustly expressed in some but not all more advanced prostate tumors. The data presented here also demonstrate that lack of CD24 did not significantly alter tumor initiation in any of the autochthonous tumor models studied. Tumor burden was independent of CD24 expression in MMTV-PyMT and TRAMP mice, and was reduced in CD24-deficient *Apc*^1572T/+^ tumors in a statistically significant manner, albeit only marginally.

In the non-transformed murine breast, CD24 is predominantly expressed by luminal epithelial cells [11]. Our data show that in luminal-like MMTV-PyMT and in metaplastic *Apc*^1572T/+^ tumors that represent a model for a subtype of human triple negative basal-like breast cancers [48], CD24 can be upregulated during early tumorigenesis (Figs 1A, 1C, 1D and 2B–2D; S1 and S2 Tables). In line with these findings, upregulation of CD24 expression has been reported for human ductal carcinoma *in situ* relative to non-transformed tissue [40]. However, while we found that CD24 expression decreased with advanced tumor progression and tumor grade both in MMTV-PyMT and in *Apc*^1572T/+^ mice (Figs 1D, 1E and 2D; S1 and S2 Tables), the expression of CD24 in late stage human invasive carcinoma of the breast is significantly increased in comparison with non-tumor tissue [40]. Our findings suggest that differences in CD24 expression in advanced luminal breast tumors exist between human patients and the MMTV-PyMT genetic mouse model. No data regarding CD24 expression are available specifically for rare human metaplastic carcinomas that would allow a direct comparison with the *Apc*^1572T/+^ model to be made. Similar to the findings here, CD24 positivity has been shown to be associated with human triple negative basal-like tumors only in early lesions [49].

In prostates from TRAMP mice, enhanced CD24 staining intensity was observed within hyperplastic lesions in comparison to adjacent non-hyperplastic regions (Fig 4B), suggesting that CD24 expression can be increased during early prostate tumorigenesis. In human prostates, CD24 expression was similarly found to be frequently present in atrophic glands and intraepithelial neoplasia, and clearly upregulated in atypical epithelia, whereas it is rare in non-neoplastic tissue and benign hyperplasias [28]. *CD24* transcripts are also significantly increased in human prostate tumors relative to benign prostate hyperplasias [50,51]. Similar to our findings, pronounced intratumoral heterogeneity of CD24 expression has also been described for human prostate carcinoma, and only a proportion of human invasive prostate carcinomas were found to be positive for CD24 [28]. Thus, CD24 expression patterns and distribution in TRAMP tumors resemble the situation in human prostate samples.

An important finding in this study is that lack of CD24 does not significantly affect tumor incidence in any of the models investigated. Similar results have been reported in carcinogen-induced urothelial tumors, where initiation of tumors in *CD24*^*-/-*^ mice was significantly delayed, but tumor incidence was not significantly different between wild-type and *CD24*^*-/-*^ mice 28 weeks after treatment with carcinogen [52]. Furthermore, while our study was in preparation, others reported that CD24 deficiency in TRAMP mice results in delayed onset of prostate tumorigenesis, but that all mice had developed prostate tumors by 5 months of age [53]. Together these observations suggest that CD24 is not essentially required for tumorigenesis in these models.

In contrast to the results reported here, CD24 deficiency resulted in resistance to chemically induced colorectal cancer, and tumor formation in *Apc*^Min^ mice that spontaneously develop intestinal tumors was almost completely prevented on a *CD24*^-/-^ background [54]. These observations suggest that CD24 can have cell type-specific effects on tumor initiation and growth, a notion supported by our finding that CD24 deficiency slightly but statistically significantly reduced tumor burden in *Apc*^1572T/+^ but not MMTV-PyMT mammary carcinomas. Cell type-specific effects of CD24 deficiency on tumor initiation and growth could conceivably be due to cell type-specific upregulation of compensatory molecules such as the CD24 paralog CD52 in *CD24*^-/-^ mice, although we note that no such compensatory upregulation of CD52 occurs in the mammary glands of *CD24*^-/-^ mice [11]. More likely, given that MMTV-PyMT mammary carcinogenesis is β-catenin independent [55] and that β-catenin can regulate CD24 expression [54], it is tempting to speculate that CD24 might be functionally relevant during APC/β-catenin-dependent tumorigenesis, which might explain why some tumor types appear to be more sensitive to CD24 deficiency than others. Furthermore, as CD24 deficiency can result in changes in immune cell subpopulations [19–21], we also cannot currently rule out a scenario in which CD24 deficiency affects the tumor microenvironment, for example by modulating the numbers of tumor promoting and suppressing immune cells, which could also conceivably have tissue-specific effects.

CD24 expression correlates with poor patient survival in a variety of human tumor types [28,29], and a functional role for CD24 in tumor progression is well established [35,37,39]. At first sight, reduced CD24 expression in later stages of MMTV-PyMT and *Apc*^1572T/+^ tumors, and absence of CD24 expression in a proportion of advanced TRAMP prostate tumors may appear contradictory with this notion. However, CD24 positivity or negativity rather than expression intensity correlated with poor survival in human prostate and breast tumor patients [28,29]. Moreover, metastasis has been suggested to be an early event during breast tumor development [56], and dissemination might therefore already take place when CD24 levels are still high. Hence, our findings that CD24 expression is reduced in but not absent from late stages of MMTV-PyMT and *Apc*^1572T/+^ tumors, and only strongly expressed in some but not all advanced TRAMP tumors, does not exclude a role for CD24 in subsequent metastasis. The number of metastases that developed on the C57BL/6 background in the tumor models used in this current study were too small to detect statistically significant differences between *CD24*^-/-^ and *CD24*^+/+^ mice. Nevertheless we note that CD24 deficiency has been reported to result in reduced metastases from urothelial tumors, but only in male mice due to androgen-dependent effects [52].

CD24 has gained attention as a potential marker for putative cancer stem cells (CSCs) that, according to the CSC-concept, initiate and drive solid tumor growth [48,57,58]. Both the presence and absence of CD24 has been ascribed as a marker of putative CSCs. Thus while CD24 positivity can define populations enriched for CSCs in human carcinomas of the pancreas [59] and ovary [60], CD24 negativity enriches for putative human breast [61] and prostate [62,63] CSCs. In breast tumors arising in MMTV-PyMT and p53^-/-^ mice, CD24 positivity is a marker for CSCs [64,65]. In spheriod-forming assays using tumor cells derived from *Apc*^1572T/+^ mice, Lin-CD29+CD24+ tumor cells gave rise to significantly more spheroids than Lin-CD29+CD24- tumor cells, suggesting that CD24-expressing cells are enriched for stemness properties [48]. Accordingly, CD24-expressing cells possess tumor initiating properties in this model [48]. If CD24 functionally contributes to the stemness properties of CSCs in these models, then loss of CD24 would be expected to have pronounced effects on tumor initiation and growth. Thus our finding that lack of CD24 does not impair tumor incidence in the MMTV-PyMT and *Apc*^1572T/+^ models (as assessed by the age at which tumors first become palpable) could suggest that CD24 may not have a functional role in determining the properties of CSCs in these models. Nevertheless we cannot rule out the possibility that *ex vivo* manipulation of the *Apc*^1572T/+^ tumor cells for the purposes of spheriod-forming and tumor initiation experiments [48] may have changed the properties of the tumor cells, as we have previously reported in other models [66].

In conclusion, our data demonstrate that while CD24 is distinctively but diversely expressed during tumor development in genetic models of breast and prostate cancer, lack of CD24 did not significantly influence tumor initiation and only partly affected tumor burden in these models. Furthermore, CD24 deficiency in *Apc*^1572T/+^ mice slightly but statistically significantly reduced tumor burden, whereas no such effect was observed in MMTV-PyMT mice, suggesting that CD24 may play different roles in different types of breast cancer. Consistently, these and other data suggest that CD24 can influence the kinetics of tumor initiation in a context-dependent manner.

## Supporting Information

S1 Table CD24 is differentially expressed in MMTV-PyMT mammary tumors, and the expression levels correlate with histopathologic appearance. Female MMTV-PyMT mice of various ages were sacrificed, and their mammary glands were cut into sections and stained with antibodies specific for CD24. A histopathologic analysis was performed and the intensity of the CD24 staining was evaluated. Score:—no staining; + moderate staining; ++ strong staining; empty cell, lesion not detected. A two-sided Fisher´s exact test was performed to test the null hypothesis "staining intensity is independent of histopathologic appearance". The null hypothesis was rejected based on a calculated p-value of 0.00035 (3x3 contingency table). Scoring was categorized into CD24 negative ("-") or CD24 positive ("+" or "++"), and two-sided Fisher´s exact tests and 2x2 contingency tables were used to perform pairwise comparisons of (i) "invasive well differentiated" vs. "invasive poorly differentiated" (p = 0.21), (ii) "preinvasive" vs. "invasive well differentiated" (p = 0.45) and (iii) "preinvasive" vs. "invasive poorly differentiated" (p = 0.015).
(DOCX)

S2 Table CD24 is differentially expressed in *Apc*^*1572/T+*^ mammary tumors, and the expression levels correlate with histopathologic appearance. Female *Apc*^*1572/T+*^ mice of various ages were sacrificed, and their mammary glands were cut into sections and stained with antibodies specific for CD24. A histopathologic analysis was performed and the intensity of the CD24 staining was evaluated. Score:—no staining; + moderate staining; ++ strong staining; empty cell, lesion not detected. A two-sided Fisher´s exact test was performed to test the null hypothesis "staining intensity is independent of histopathologic appearance". The null hypothesis was rejected based on a calculated p-value of 0.0000002 (2x2 contingency table).
(DOCX)

S3 Table CD24 is heterogenously expressed in TRAMP prostate tumors, and the expression levels differ between distinct histopathologic appearances. Male TRAMP mice of various ages were sacrificed, and their prostate glands were cut into sections and stained with antibodies specific for CD24. A histopathologic analysis was performed and the intensity of the CD24 staining was evaluated. Score:—no staining; + moderate staining; ++ strong staining; empty cell, lesion not detected. A two-sided Fisher´s exact test was performed to test the null hypothesis "staining intensity is independent of histopathologic appearance". The null hypothesis was rejected based on a calculated p-value of 0,0000007 (3x4 contingency table). Scoring was categorized into CD24 negative ("-") or CD24 positive ("+" or "++"), and two-sided Fisher´s exact tests and 2x2 contingency tables were used to perform pairwise comparisons of (i) "invasive well differentiated" vs. "invasive poorly differentiated" (p = 0.006), (ii) "preinvasive" vs. "invasive well differentiated" (p = 0.015) and (iii) "preinvasive" vs. "invasive poorly differentiated" (p = 0.45).
(DOCX)
