# Supplementary material for: CD24 Is Not Required for Tumor Initiation and Growth in Murine Breast and Prostate Cancer Models
Source: PLoS One. 2016 Mar 15;11(3):e0151468. doi: 10.1371/journal.pone.0151468 (PMC4792398; doi:10.1371/journal.pone.0151468)
Supplement: S1 Table — Female MMTV-PyMT mice of various ages were sacrificed, and their mammary glands were cut into sections and stained with antibodies specific for CD24. A histopathologic analysis was performed and the intensity of the CD24 staining was evaluated. Score:—no staining; + moderate staining; ++ strong staining; empty cell, lesion not detected. A two-sided Fisher´s exact test was performed to test the null hypothesis "staining intensity is independent of histopathologic appearance". The null hypothesis was rejected based on a calculated p-value of 0.00035 (3x3 contingency table). Scoring was categorized into CD24 negative ("-") or CD24 positive ("+" or "++"), and two-sided Fisher´s exact tests and 2x2 contingency tables were used to perform pairwise comparisons of (i) "invasive well differentiated" vs. "invasive poorly differentiated" (p = 0.21), (ii) "preinvasive" vs. "invasive well differentiated" (p = 0.45) and (iii) "preinvasive" vs. "invasive poorly differentiated" (p = 0.015). (DOCX) [file pone.0151468.s001.docx]

**S1 Table. CD24 is differentially expressed in MMTV-PyMT mammary tumors, and the expression levels correlate with histopathologic appearance.**

| **Animal #** | **Preinvasive** | **Invasive well differentiated** | **Invasive poorly differentiated** |
| --- | --- | --- | --- |
| 1 |  |  | + |
| 2 | + |  |  |
| 3 |  | ++ | - |
| 4 |  | ++ | - |
| 5 | ++ | ++ |  |
| 6 | + |  |  |
| 7 | ++ |  |  |
| 8 |  |  | - |
| 9 | + |  |  |
| 10 | ++ |  |  |
| 11 |  | ++ | - |
| 12 |  | - |  |
