# Supplementary material for: CD24 Is Not Required for Tumor Initiation and Growth in Murine Breast and Prostate Cancer Models
Source: PLoS One. 2016 Mar 15;11(3):e0151468. doi: 10.1371/journal.pone.0151468 (PMC4792398; doi:10.1371/journal.pone.0151468)
Supplement: S2 Table — Female Apc1572/T+ mice of various ages were sacrificed, and their mammary glands were cut into sections and stained with antibodies specific for CD24. A histopathologic analysis was performed and the intensity of the CD24 staining was evaluated. Score:—no staining; + moderate staining; ++ strong staining; empty cell, lesion not detected. A two-sided Fisher´s exact test was performed to test the null hypothesis "staining intensity is independent of histopathologic appearance". The null hypothesis was rejected based on a calculated p-value of 0.0000002 (2x2 contingency table). (DOCX) [file pone.0151468.s002.docx]

**S2 Table. CD24 is differentially expressed in *Apc^1572/T+^*** **mammary tumors, and the expression levels correlate with histopathologic appearance.**

| **Animal #** | **Invasive well differentiated** | **Invasive poorly differentiated** |
| --- | --- | --- |
| 1 | ++ | - |
| 2 | ++ | - |
| 3 | ++ | - |
| 4 | ++ |  |
| 5 | ++ | - |
| 6 | ++ | - |
| 7 | ++ | - |
| 8 | ++ | - |
| 9 | ++ | - |
| 10 | ++ | - |
| 11 | ++ | - |
| 12 | ++ |  |
| 13 | ++ | - |
| 14 | ++ |  |
