# Supplementary material for: CD24 Is Not Required for Tumor Initiation and Growth in Murine Breast and Prostate Cancer Models
Source: PLoS One. 2016 Mar 15;11(3):e0151468. doi: 10.1371/journal.pone.0151468 (PMC4792398; doi:10.1371/journal.pone.0151468)
Supplement: S3 Table — Male TRAMP mice of various ages were sacrificed, and their prostate glands were cut into sections and stained with antibodies specific for CD24. A histopathologic analysis was performed and the intensity of the CD24 staining was evaluated. Score:—no staining; + moderate staining; ++ strong staining; empty cell, lesion not detected. A two-sided Fisher´s exact test was performed to test the null hypothesis "staining intensity is independent of histopathologic appearance". The null hypothesis was rejected based on a calculated p-value of 0,0000007 (3x4 contingency table). Scoring was categorized into CD24 negative ("-") or CD24 positive ("+" or "++"), and two-sided Fisher´s exact tests and 2x2 contingency tables were used to perform pairwise comparisons of (i) "invasive well differentiated" vs. "invasive poorly differentiated" (p = 0.006), (ii) "preinvasive" vs. "invasive well differentiated" (p = 0.015) and (iii) "preinvasive" vs. "invasive poorly differentiated" (p = 0.45). (DOCX) [file pone.0151468.s003.docx]

**S3 Table. CD24 is heterogenously expressed in TRAMP prostate tumors, and the expression levels differ between distinct histopathologic appearances.**

| **Animal #** | **Preinvasive** | **Invasive well differentiated** | **Invasive poorly differentiated** |
| --- | --- | --- | --- |
| 1 |  |  | **-** |
| 2 |  | **++** |  |
| 3 | **-** |  | **++** |
| 4 |  | **++** |  |
| 5 |  |  | **++** |
| 6 |  | **+** |  |
| 7 |  |  | **++** |
| 8 |  |  | **+** |
| 9 | **++** |  |  |
| 10 | **+** |  |  |
| 11 | **+** |  |  |
| 12 | **++** |  |  |
| 13 |  | **-** |  |
| 14 |  |  | **+** |
| 15 | **-** |  |  |
| 16 | **+** |  |  |
| 17 |  | **++** |  |
| 18 | **+** |  |  |
| 19 | **-** |  |  |
| 20 | **-** |  | **++** |
| 21 | **++** |  |  |
| 22 | **++** |  |  |
| 23 | **-** |  |  |
| 24 | **+** |  |  |
| 25 | **+** |  |  |
| 26 | **-** | **-** |  |
| 27 | **-** | **-** |  |
| 28 | **++** |  |  |
| 29 | **-** | **-** |  |
| 30 | **+** |  |  |
| 31 | **+** |  |  |
| 32 |  |  |  |
| 33 | **+** | **-** |  |
| 34 |  | **-** |  |
| 35 |  | **-** |  |
| 36 |  |  | **++** |
| 37 |  | **-** |  |
| 38 |  | **-** |  |
| 39 | **++** |  |  |
| 40 |  |  |  |
| 41 | **+** |  |  |
| 42 |  | **-** |  |
| 43 | **-** |  |  |
| 44 |  | **-** |  |
| 45 |  |  | **-** |
| 46 | **++** |  |  |
| 47 | **+** |  |  |
| 48 | **+** |  |  |
| 49 | **-** |  | **++** |
| 50 | **-** |  | **++** |
| 51 |  | **-** |  |
| 52 |  | **-** |  |
